# Dissecting the role of glutathione biosynthesis in *Plasmodium falciparum*

**DOI:** 10.1111/j.1365-2958.2011.07933.x

**Published:** 2012-01

**Authors:** Eva-Maria Patzewitz, Eleanor H Wong, Sylke Müller

**Affiliations:** Wellcome Trust Centre for Molecular Parasitology, Institute of Infection, Immunity & Inflammation, College of Medical, Veterinary and Life Sciences, University of Glasgow120 University Place, Glasgow G12 8TA, UK.

## Abstract

Glutathione (γ-glutamylcysteinyl-glycine, GSH) has vital functions as thiol redox buffer and cofactor of antioxidant and detoxification enzymes. *Plasmodium falciparum* possesses a functional GSH biosynthesis pathway and contains mM concentrations of the tripeptide. It was impossible to delete in *P. falciparum* the genes encoding γ-glutamylcysteine synthetase (γGCS) or glutathione synthetase (GS), the two enzymes synthesizing GSH, although both gene loci were not refractory to recombination. Our data show that the parasites cannot compensate for the loss of GSH biosynthesis via GSH uptake. This suggests an important if not essential function of GSH biosynthesis pathway for the parasites. Treatment with the irreversible inhibitor of γGCS L-buthionine sulfoximine (BSO) reduced intracellular GSH levels in *P. falciparum* and was lethal for their intra-erythrocytic development, corroborating the suggestion that GSH biosynthesis is important for parasite survival. Episomal expression of *γgcs* in *P. falciparum* increased tolerance to BSO attributable to increased levels of γGCS. Concomitantly expression of glutathione reductase was reduced leading to an increased GSH efflux. Together these data indicate that GSH levels are tightly regulated by a functional GSH biosynthesis and the reduction of GSSG.

## Introduction

All living organisms need to maintain an adequate intracellular redox environment. In most organisms glutathione (γ-glutamylcysteinyl-glycine; GSH) represents the major low molecular weight thiol and its intracellular concentration varies between 1 and 10 mM with the majority being in its reduced form. It serves as thiol redox buffer that guarantees maintenance of the intracellular reducing environment (Meister and Anderson, 1983). GSH also acts as cofactor for enzymes such as glutathione peroxidases, glutathione-*S*-transferases (GST) and glyoxylases (Meister, 1983; Meister and Anderson, 1983; Becker *et al*., 2003). All three enzymatic systems are involved in protecting cells against reactive oxygen species, toxic metabolic intermediates and xenobiotics (Meister and Anderson, 1983; Meister, 1988). In addition, GSH is also known as non-enzymatic antioxidant either alone or in conjunction with vitamin C and vitamin E (Linster and Van Schaftingen, 2007, Galli and Azzi, 2010). GSH is synthesized *de novo* by two consecutive ATP-dependent enzymatic reactions catalysed by γ-glutamylcysteine synthetase (γGCS) and glutathione synthetase (GS) (Meister and Anderson, 1983; Meister, 1988). Mammalian γGCS is composed of a catalytic and regulatory subunit, which are distinct gene products (Huang *et al*., 1993a,b). The enzymes from bacteria, fungi and the protozoan parasites *Trypanosoma brucei* and *Plasmodium falciparum* consist only of the catalytic subunit (Coblenz and Wolf, 1995; Lueder and Phillips, 1996; Griffith and Mulcahy, 1999; Lüersen *et al*., 1999; Kelly *et al*., 2002; Baek *et al*., 2004), suggesting that the way GSH biosynthesis is regulated differs fundamentally between mammals and these microorganisms.

The important and versatile functions of GSH suggest that the tripeptide is vital for most organisms including the human malaria parasite *P. falciparum* (Becker *et al*., 2003). *P. falciparum* is the causative agent of the most severe form of human malaria and the infection with the protozoan parasite leads to approximately 1 million human deaths per annum. Apart from being a serious public health problem, malaria is a major economic burden in tropical and subtropical countries, especially in sub-Saharan Africa. The malaria parasite possesses a highly developed antioxidant system to help it cope with the pro-oxidant environment it encounters during its development in the mammalian and insect hosts (Müller *et al*., 2001; Becker *et al*., 2003; 2004; Müller, 2004). The GSH system of the parasite consists of the GSH biosynthesis pathway, glutathione reductase (GR) to maintain an adequate ratio of reduced GSH to oxidized glutathione disulphide (GSSG), glyoxalases I and II, glutaredoxins and a single GST (Farber *et al*., 1996; Lüersen *et al*., 1999; Rahlfs *et al*., 2001; Harwaldt *et al*., 2002; Liebau *et al*., 2002; Meierjohann *et al*., 2002a; Akoachere *et al*., 2005). GSH also reduces thioredoxin-disulphides and thus links the two main antioxidant and redox systems operating in the parasites (Kanzok *et al*., 2000; Krnajski *et al*., 2001; Becker *et al*., 2003; 2004; Müller, 2004). In addition, both redox systems provide reducing equivalents for the reaction catalysed by ribonucleotide reductase and thus are important for DNA synthesis and cell proliferation (Becker *et al*., 2004; Müller, 2004).

The parasites possess the two enzymes γGCS and GS to generate the tripeptide *de novo* from the amino acids glutamate, cysteine and glycine (Lüersen *et al*., 1999; Meierjohann *et al*., 2002a) and, provided a suitable supply of the amino acids, are independent of a source of GSH from their host. Inhibition of the biosynthetic pathway by the γGCS inhibitor L-buthionine sulfoximine (BSO) is lethal for *P. falciparum* during intra-erythrocytic growth, implying that the parasites rely on a functional GSH biosynthesis (Lüersen *et al*., 2000). Therefore it is surprising that the related rodent malaria parasite *Plasmodium berghei* does not rely on its endogenous GSH biosynthesis during the development in the mammalian host. The deletion of the *P. berghei γgcs* gene affects parasite growth in the red blood cells (RBC) only marginally. The mutant parasites still contain low but apparently adequate levels of GSH despite the lack of γGCS function, presumably because they take up the tripeptide from their host by a yet unidentified mechanism. The *P. berghei γgcs* null mutants depend however on a functional GSH biosynthesis during their sexual development, possibly because the demand for the tripeptide is increased during these developmental stages or the insect host does not supply sufficient GSH to complement for a loss of GSH biosynthesis (Vega-Rodriguez *et al*., 2009). Similarly, it was recently shown that *gr* is not essential in *P. berghei* during intra-erythrocytic growth but is crucial for the development of the insect stages of the parasites (Buchholz *et al*., 2010; Pastrana-Mena *et al*., 2010). Interestingly, double null mutants for *γgcs* and *gr* in *P. berghei* are non-viable (Buchholz *et al*., 2010), indicating that the loss of either of these genes renders the other indispensable in RBC stages, presumably because a residual concentration of GSH needs to be maintained.

Our study provides evidence that, in contrast to the situation in *P. berghei*, GSH biosynthesis is vital in the human malaria parasite *P. falciparum* for blood stage development during *in vitro* growth. The reason for this is that *P. falciparum* is not taking up substantial amounts of GSH from the external environment but loose large amounts of GSSG through significant efflux (Atamna and Ginsburg, 1997; Ayi *et al*., 1998; Lüersen *et al*., 2000). Probing the role of GSH biosynthesis by using BSO, the specific inhibitor of γGCS (Griffith, 1982), and by overexpressing γGCS, we further demonstrate that *P. falciparum* GSH metabolism is tightly regulated by both GSH biosynthesis and GSSG reduction.

## Results

### The *γgcs* and *gs genes* are not disrupted by single homologous recombination

**Figure 1:**
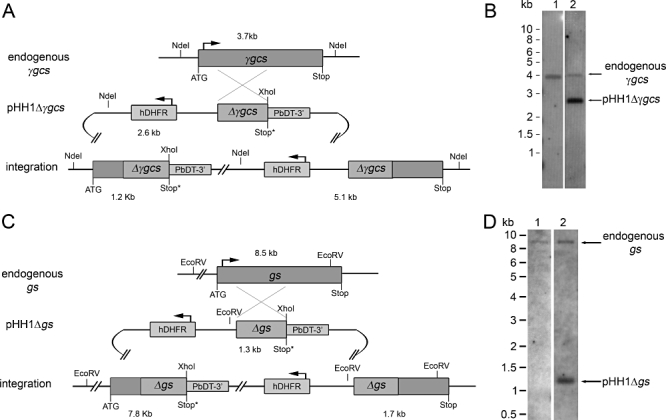
Gene disruption of *γgcs* and *gs*. A. Schematic representation (not to scale) of the endogenous *γgcs* locus, the pHH1Δ*γgcs* plasmid and the recombined *γgcs* locus following single cross-over recombination and integration of the plasmid. NdeI and XhoI restriction sites and the sizes of the resulting fragments are indicated. The plasmid contains a fragment of *γgcs* truncated at both 5′ and 3′ ends (Δ*γgcs*) and a human *dihydrofolate reductase* selectable marker cassette (hDHFR). Recombination between plasmid and endogenous locus leads to a pseudo-diploid locus with two non-functional copies of the gene, one truncated at the 3′ end upstream and one truncated at the 5′ end downstream of the hDHFR cassette. B. Southern blot analysis of DNA isolated from parasites transfected with pHH1Δ*γgcs* after two drug selection cycles (lane 2) in comparison with non-transfected wild-type parasites (lane 1). No bands indicating integration at 1.2 kb and 5.2 kb are visible in lane 2, only the 2.6 kb plasmid band and the 3.7 kb band corresponding to the endogenous *γgcs* locus. C. Schematic representation of the endogenous *gs* locus, the pHH1Δ*gs* plasmid and the recombined *gs* locus following integration of the plasmid by single cross-over. Restriction sites for EcoRV and XhoI are indicated as well as the sizes of the expected DNA fragments. The pHH1Δ*gs* plasmid contains a insert homologous to *gs* but lacking the 5′ and 3′ ends (Δ*gs*) and a hDHFR selectable marker cassette. Integration of the plasmid causes the formation of a pseudo-diploid locus containing two non-functional copies of *gs*, the upstream copy being truncated at the 3′ end and the downstream copy being truncated at the 5′ end. D. Southern blot analysis of DNA isolated form parasite lines transfected with pHH1Δ*gs* after two drug selection cycles (lane 2) in comparison with wild-type parasites (lane 1). No integration specific bands at 7.8 kb and 1.3 kb are visible in the DNA from the transfected parasite line. The endogenous *gs* specific band is visible in both wild-type and transfected parasites at 8.5 kb. In addition, a 1.3 kb band corresponding to the plasmid is visible in the transfected parasites.

Attempts to disrupt the *γgcs* or *gs* gene loci were unsuccessful using constructs generated in the pHH1 transfection plasmid (Reed *et al*., 2000). The constructs contained either an 800 bp fragment of the *γgcs* gene truncated at the 5′ and 3′ end (pHH1Δ*γgcs*) or a 1004 bp fragment of *gs* lacking the 5′ and 3′ end of the *gs* gene (pHH1Δ*gs*). Both constructs lacked the ATG start codon and possessed an artificial stop codon (Fig. 1). It was expected that upon targeting the respective gene loci, the endogenous genes would be disrupted to result in the formation of pseudo-diploid loci with two truncated copies of the respective genes (Fig. 1A and C). WR99210 resistant parasites were observed in Giemsa stained thin smears 4 weeks after transfection. Parasites were taken through so-called ‘drug selection cycles’, where the selectable drug was withdrawn for a period of 3 weeks followed by a short period with addition of drug until the parasitaemia reached 5% again. Genomic DNA was isolated in each drug selection cycle and subjected to diagnostic digestions followed by Southern blot analyses. The parasite line transfected with pHH1Δ*γgcs* showed the presence of the plasmid (2.6 kb) and of the endogenous gene (3.7 kb). DNA fragments indicating the targeting of the gene locus were not observed (Fig. 1B). This suggests that the *γgcs* gene locus is either not targeted by the transfection plasmid or that the gene is essential under the *in vitro* culture conditions.

A similar approach was taken for the disruption of the *gs* gene (Fig. 1C and D). The diagnostic Southern blot after transfection of parasites with pHH1Δ*gs* probed with the *gs* specific probe should detect a 8.5 kb endogenous *gs* fragment and the expected size of the restricted transfection plasmid is 1.3 kb with this probe. Upon recombination of the plasmid, the expected DNA fragments detectable on the blot should be 1.7 kb and 7.8 kb respectively (Fig. 1C). The endogenous *gs* gene was detected in wild-type and transfected parasite lines and the transfected parasite line also contained the diagnostic plasmid band when DNA was isolated after drug selection cycle 4 (Fig. 1D). However, no DNA fragments indicative of a *gs* gene disruption were detected suggesting that even after four drug selection cycles the plasmid had not targeted the gene locus of interest.

### Knockouts of *γgcs* and *gs* are not possible by double homologous recombination

**Figure 2:**
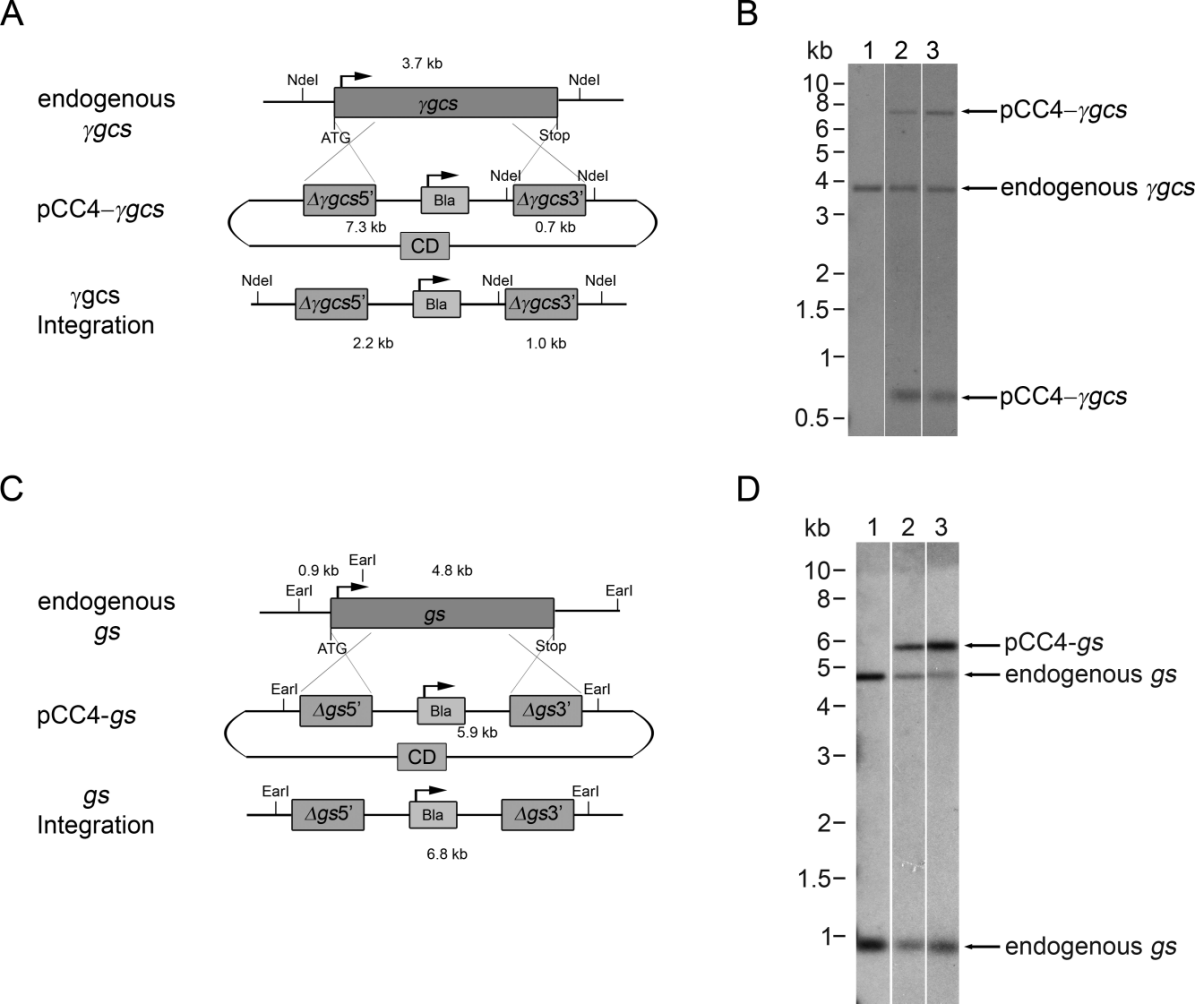
Gene replacement of *γgcs* and *gs*. A. Schematic representation of the endogenous *γgcs* locus, the pCC4-*γgcs* knockout construct and the recombined *γgcs* locus following integration. NdeI restriction sites for diagnostic Southern analyses and sizes of the expected DNA fragments are indicated. The positive selectable marker blasticidin-*S*-deaminase (Bla) is flanked by two regions homologous to the 5′ (Δ*γgcs*5′) and 3′ (Δ*γgcs*3′) end of *γgcs* and replaces part of the target gene after recombination. The negative selectable marker cytosine deaminase (CD) is lost upon double cross-over recombination. B. Southern blot of parasites transfected with pCC4-*γgcs* in comparison with non-transfected wild-type parasites (lane 1). No integration was observed after three drug selection cycles and the plasmid was still present (7.3 kb and 0.7 kb bands, lane 2). 5-FC resistant parasites were obtained after drug selection cycle 3, but the plasmid was still present (lane 3). C. Schematic diagram of the endogenous *gs* locus, the pCC4-*gs* knockout plasmid and the recombined *gs* locus following double cross-over between the 5′ homologous fragment (Δ*gs*5′) and 3′ fragment (Δ*gs*3′) of pCC4-*gs* and the endogenous locus. EarI restriction sites used for Southern analyses and diagnostic DNA fragment sizes are indicated. D. Southern analyses of pCC4-*gs* transfected parasites. Lane 1 contains digested DNA from wild-type parasites. Lane 2 shows DNA isolated from parasites in transfection cycle 3. In lane 3 DNA of 5-FC resistant parasites was loaded. The 0.9 kb and 4.8 kb bands diagnostic for the endogenous *gs* locus are visible in all three lanes. The 5.9 kb plasmid band is present in transfected parasites (lanes 2 and 3). No bands indicating plasmid integration into the *gs* gene locus are visible.

We further attempted to replace the *γgcs* and *gs* genes using knockout constructs based on the plasmid pCC4. This plasmid allows for positive and negative selection and confers double cross-over recombination (Braks *et al*., 2006; Maier *et al*., 2006) (Fig. 2). To support parasite growth in the absence of GSH biosynthesis the medium was supplemented with 1 mM GSH to compensate for the loss of *γgcs* or *gs*. This concentration of GSH was chosen because it had no adverse effects on parasite growth and it is well above the physiological levels of the tripeptide in human plasma and serum, which has been reported to be between 0.3 and 20 µM (Wendel and Cikryt, 1980; Jeevanandam *et al*., 2000; Scibior *et al*., 2008). Parasites resistant to blasticidin were identified 4 weeks after transfection. After each drug selection cycle parasites were subjected to negative selection with 5-fluorocytosine (5-FC) to select against episomal maintenance of the transfection plasmid. Genomic DNA of positive and negative selected parasites was isolated and analysed by diagnostic Southern blotting (Fig. 2). The Southern blots of parasites transfected with pCC4-*γgcs* revealed that after three selection cycles the endogenous gene (3.7 kb) and the plasmid (0.7 kb and 7.3 kb) were present (Fig. 2A and B; lane 2). Even in 5-FC resistant parasites gene-specific and plasmid-specific bands were still detected, suggesting that the parasites had developed resistance against 5-FC without loss of the episomal plasmid (Fig. 2A and B; lane 3). Similar results were obtained when analysing parasites transfected with pCC4-*gs*. The Southern blot shows the bands diagnostic for endogenous *gs* (0.9 kb and 4.8 kb) and the plasmid-specific band of 5.9 kb in both untreated and 5-FC-treated parasites after selection cycle 3 (Fig. 2C and D). These data imply that both gene loci cannot be replaced by the knockout constructs. This is again either due to refractory gene loci or suggests that the genes are important for parasite survival during development in the RBC even when the medium has been supplemented with 1 mM GSH.

### Targeting of *γgcs* and *gs* loci is possible

**Figure 3:**
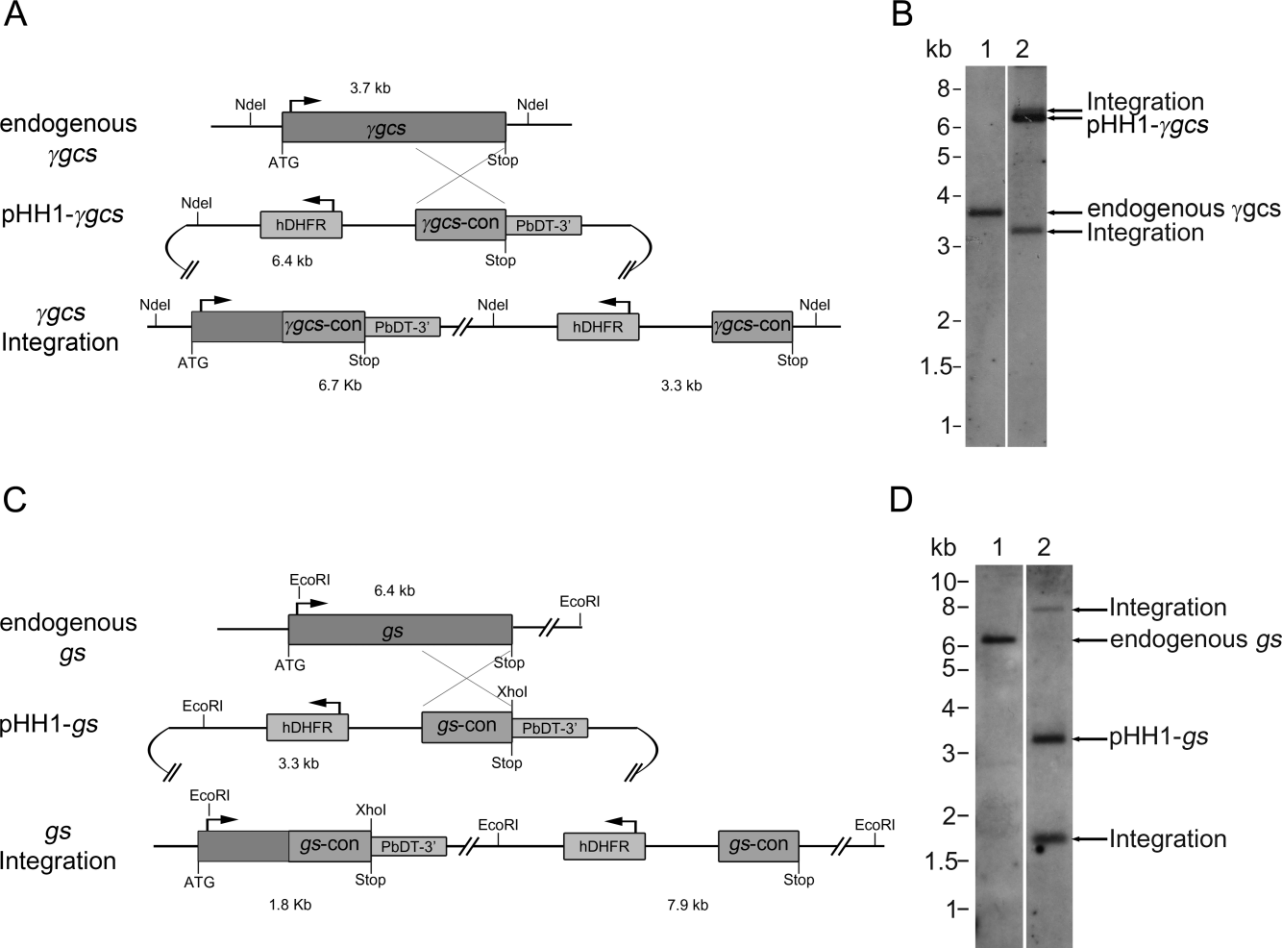
Targeting of *γgcs* and *gs* loci is possible. A. Schematic diagram of the endogenous *γgcs* locus, the pHH1-*γgcs* plasmid and the recombined *γgcs* locus following single cross-over recombination between the plasmid and the 3′ region of *γgcs*. The plasmid contains a human dihydrofolate reductase selectable marker (hDHFR) cassette, a region homologous to the 3′ end of *γgcs* (*γgcs*-con) allowing for recombination with the target gene and an artificial 3′ UTR (*P. berghei* dihydrofolate reductase/thymidylate synthase 3′ UTR, PbDT-3′). NdeI restriction sites and sizes of the resulting diagnostic DNA fragments are indicated. B. Southern blot of wild-type parasites (lane 1) in comparison with a representative clone (lane 2) in which the *γgcs* locus has been targeted by the pHH1-*γgcs* control plasmid. The 3.7 kb endogenous *γgcs* band was only visible in the wild type (lane 1). In the cloned parasites (lane 2) two bands (3.3 kb and 6.7 kb) diagnostic for integration of the plasmid into the *γgcs* gene locus were detected. The pHH1-*γgcs* plasmid (6.4 kb) was still present in the cloned parasite line. C. Schematic diagram of the *gs* locus, the pHH1-*gs* plasmid and the recombined *gs* locus following integration. The plasmid contains an hDHFR selectable marker cassette and a homologous fragment to the 3′ end of *gs* (*gs*-con) with an artificial downstream 3′ UTR (PbDT-3′). EcoRI and XhoI restriction sites used for diagnostic digest and Southern blotting are indicated as well as the sizes of the resulting DNA fragments. D. Southern blot analysis of wild-type parasites (lane 1) in comparison with a representative cloned parasite line (lane 2) bearing the recombined *gs* locus following integration of pHH1-*gs*. The 6.4 kb band corresponding to endogenous *gs* was only detectable in the DNA isolated from wild-type parasites. In the cloned parasite line two bands specific for integration in the *gs* gene locus of 1.8 kb and 7.9 kb were visible (lane 2). The plasmid (3.3 kb) was still detectable in the cloned parasite line.

In order to analyse whether the *γgcs* or *gs* loci were refractory to recombination parasites were transfected with pHH1-based knock-in constructs (Crabb *et al*., 2004). The constructs lacked the ATG start codon but contained the C-terminal part of either gene as outlined in the *Experimental procedures*. Parasites were grown without addition of exogenous GSH. In parasites transfected with pHH1-*γgcs* the gene locus was targeted after one selection cycle and parasites were cloned by limiting dilution. Genotypes of the clones were verified by Southern blotting (Fig. 3A and B). The endogenous *γgcs* band (3.7 kb) was no longer present while the two bands characteristic for integration of the plasmid into the *γgcs* gene locus (3.3 kb and 6.7 kb) were detected as shown for a representative clone (Fig. 3B, lane 2). An additional band represents the pHH1-*γgcs* plasmid still present in the cloned parasite line.

Similarly the *gs* locus was targeted by the pHH1-*gs* construct after two selection cycles and the cloned parasites were analysed by Southern blotting (Fig. 3C and D). The diagnostic band for endogenous *gs* (6.4 kb) disappeared while those specific for the integration event into the *gs* gene locus (1.8 kb and 7.9 kb) as well as the plasmid pHH1-*gs* (3.3 kb) were detected in the representative clone (Fig. 3D).

Parasites were also co-transfected with the pCC4-γ*gcs* plasmid and an episomal expression plasmid pCHD-*γgcs(HA)_3_* to achieve the knockout of the *γgcs* gene in the presence of an episomal copy of the gene. This method has previously been used successfully for the knockout of other essential genes (Krnajski *et al*., 2002; Dorin-Semblat *et al*., 2008; Reininger *et al*., 2009). However, this attempt was also unsuccessful because the two transfected plasmids appeared to recombine after transfection, which made a meaningful analysis of the transfected parasite lines impossible (Fig. S1).

These data show that both gene loci are not refractory to recombination and strongly suggest that they are not targeted by the various knockout constructs because the loss of γ*gcs* or *gs* has negative effects on *P. falciparum* growth during RBC development *in vitro*.

### Uptake of GSH

**Figure 4:**
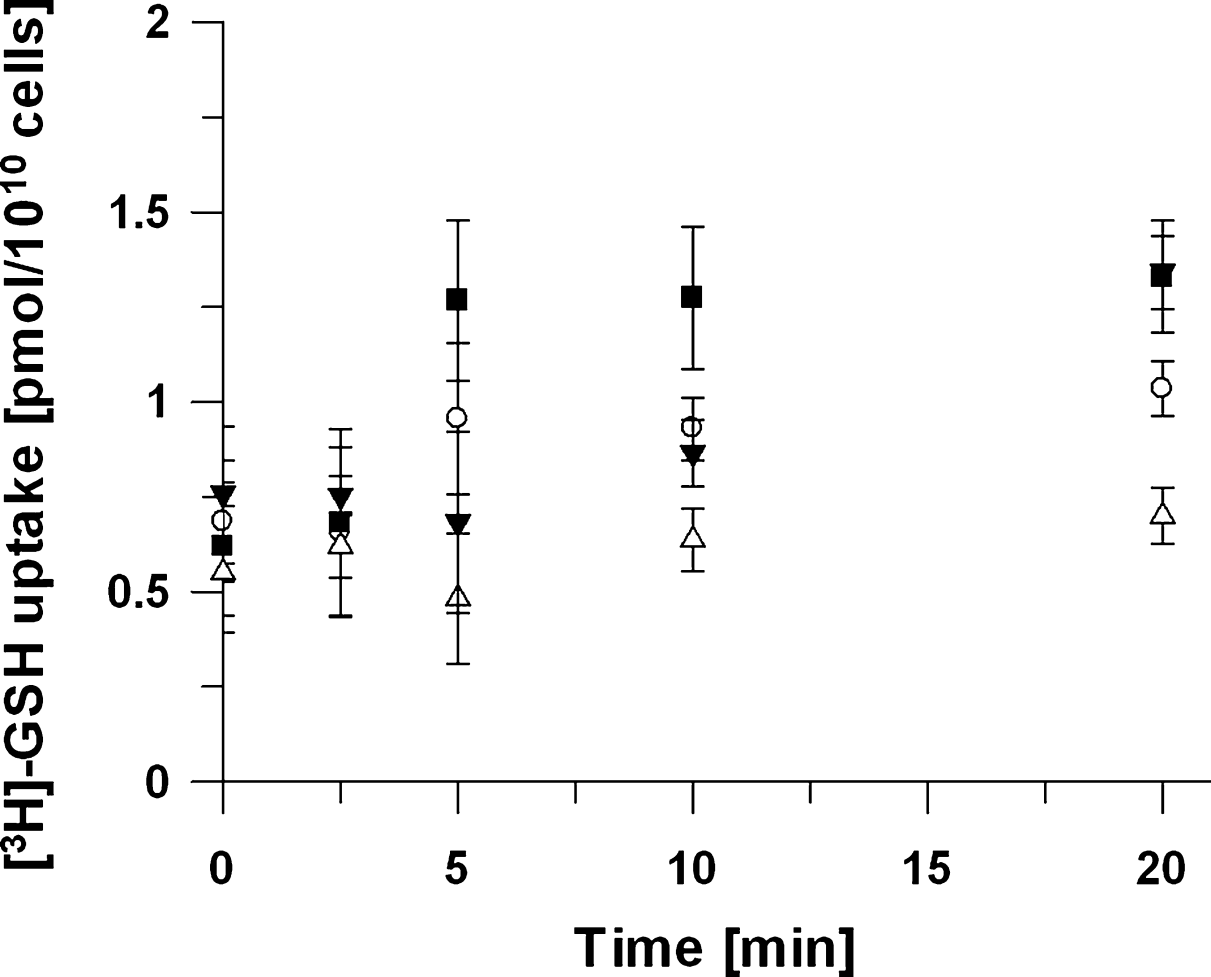
Uptake of [^3^H]-GSH. Uptake of [^3^H]-GSH was compared in uninfected (○) and purified trophozoite-infected RBC (▪). Cells were incubated with 24 nM [^3^H]-GSH and at indicated time points aliquots were withdrawn and the cell-associated label was determined. The amount of [^3^H]-GSH associated with either uninfected RBC or infected RBC did not significantly increase over 20 min (*P* > 0.05, unpaired Student *t*-test). The amount of [^3^H]-GSH taken up by MACS purified infected RBC after 20 min was not significantly higher than in the control RBC treated in the same way (*P* > 0.05, unpaired Student's *t*-test). An excess of unlabelled GSH (3 mM) did not significantly inhibit the amount of [^3^H]-GSH associated with purified trophozoite infected RBC (▾) but lowered the amount of [^3^H]-GSH associated with uninfected RBC (Δ) over 20 min. Data represent means of four independent determinations ± SEM.

One reason for the failure to generate *P. falciparum γgcs* and *gs* null mutants may be that the parasites are dependent on their endogenous GSH biosynthesis rather than being able to take up sufficient exogenous GSH. Previous studies investigating GSH uptake into *P. falciparum* are conflicting, one suggesting that GSH cannot be taken up into *P. falciparum* (Atamna and Ginsburg, 1997), while another reports that the parasites are able to obtain GSH from their environment albeit at low amounts (Raj *et al*., 2009). Our data demonstrate that *Plasmodium*-infected erythrocytes take up 1–1.5 pmol/10^10^ cells of [^3^H]-GSH in 20 min. This is only marginally higher than the amount of [^3^H]-GSH that enters uninfected RBC and this difference is not statistically significant (Student's unpaired *t*-test, *P* > 0.05) (Fig. 4). In the presence of an excess of unlabelled GSH the uptake of [^3^H]-GSH was similar to that determined with [^3^H]-GSH only (Fig. 4). These data indicate that GSH is not taken up by the parasitized RBC at significant rates and suggests that the rate of uptake is not sufficiently high to compensate for the disruption of the genes encoding γGCS and GS. Uptake of [^3^H]-GSH into the parasite was also investigated in a longer-term experiment where parasites were incubated with 24 nM [^3^H]-GSH for 2 or 4 h respectively. The increase in [^3^H]-GSH in the saponin-isolated parasites over this period of time suggests that GSH can enter the parasites at low rates (Fig. S2).

### Elevated levels of γGCS in *P. falciparum* confer resistance to BSO

**Figure 5:**
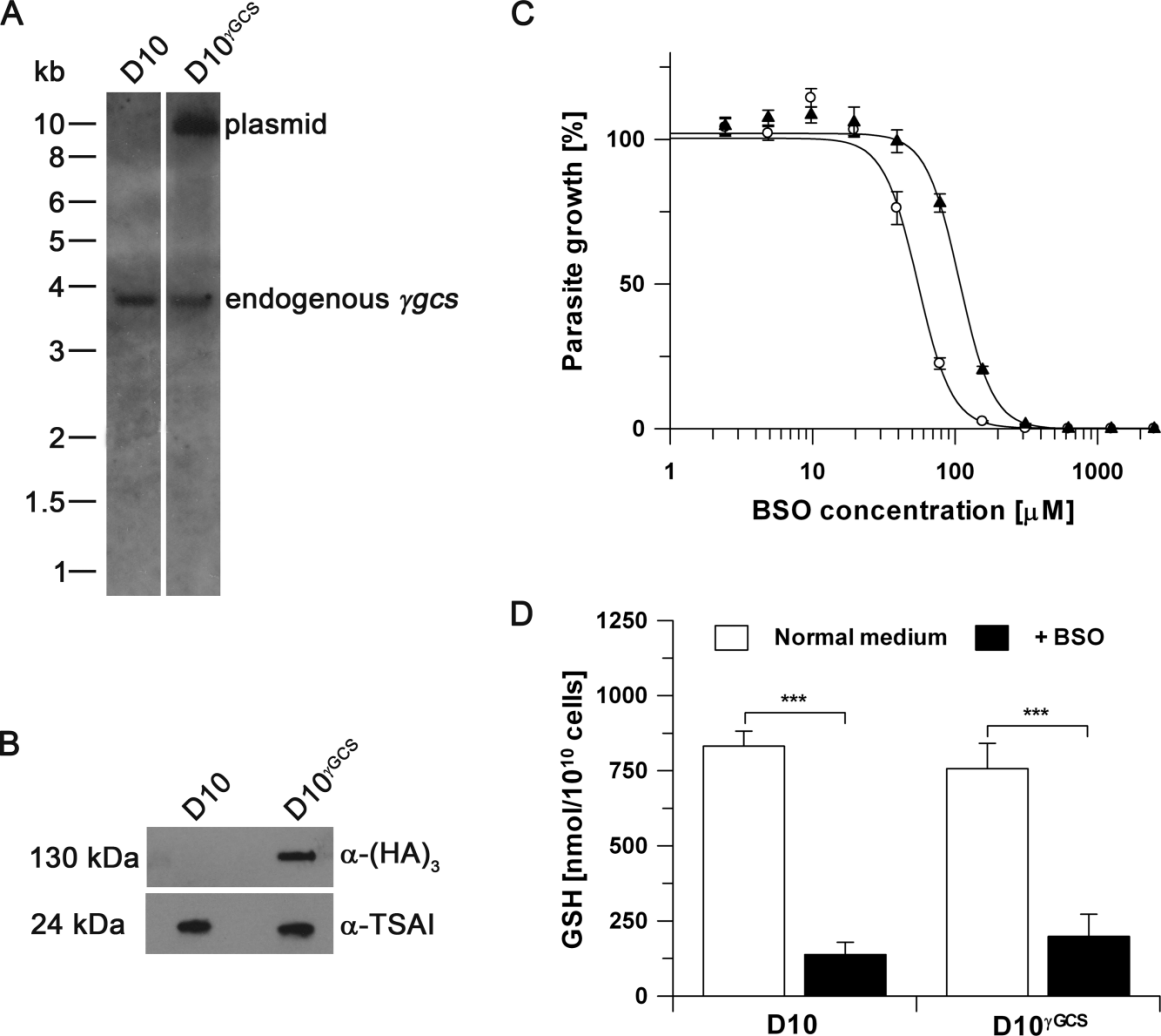
Episomal expression of γGCS-(HA)_3_ leads to increased BSO resistance. A. Southern blot analysis of wild-type D10 parasites (lane 1) in comparison with D10^γGCS^ transfected with expression plasmid pCHD-*γgcs(HA)_3_* (lane 2). The 3.9 kb band corresponding to endogenous *γgcs* and the 9.9 kb band diagnostic for the plasmid are indicated. B. Western blot analysis with anti-(HA)_3_ antibody (mouse, 1:1000) confirmed expression of γGCS-(HA)_3_ in D10^γGCS^. As a loading control an anti-*P. falciparum* 1-Cys peroxiredoxin antibody (α-TSAI, rabbit 1:120 000) was used. C. The BSO IC_50_ for wild-type D10 (○) was significantly lower (58.0 ± 2.2 µM) than for D10^γGCS^ (▴, 111.2 ± 11.1 µM) parasites expressing γGCS-(HA)_3_ (*P* < 0.001, unpaired Student's *t*-test). Data represent means of three independent determinations, each done in duplicate. Error bars represent SD. D. GSH levels in D10 and D10^γGCS^ under normal growth conditions and after incubation with 50 µM BSO for 2 h. Results represent means ± SEM of 3 to 5 independent determinations. There were no significant differences of GSH levels found between D10 (832 ± 50 nmol/10^10^ cells) and D10^γGCS^ parasites (757 ± 85 nmol/10^10^ cells) grown in normal culture medium. Exposure to BSO significantly decreased GSH levels in both D10 and D10^γGCS^ to 138 ± 41 and 199 ± 74 nmol/10^10^ cells respectively. Statistical analyses were performed using one-way ANOVA and Newman–Keuls post test (***P < 0.001).

We further probed whether GSH biosynthesis is important for *P. falciparum* RBC development using the irreversible suicide inhibitor of γGCS, BSO (Griffith, 1982). Previous studies have not answered the question whether the lethal effect of BSO is attributable to inhibition of host cell γGCS or the parasite enzyme (Lüersen *et al*., 2000). Therefore a *P. falciparum* line was generated that expressed γGCS C-terminally (HA)_3_-tagged from an episome (Fig. 5). The mutant parasite line was named D10^γGCS^. Presence of the episome and expression of the (HA)_3_-tagged protein were verified by Southern and Western blotting (Fig. 5A and B). The Southern blot of the transfected parasites shows the presence of the endogenous gene (3.7 kb) and the plasmid pCHD-*γgcs(HA)_3_* (9.9 kb) (Fig. 5A) and the Western blot shows expression of the (HA)_3_-tagged protein in D10^γGCS^ (130 kDa; Fig. 5B). In agreement with an overexpression of γGCS, the enzymatic activity in the transgenic parasite line was increased from 2.9 ± 1.1 nmol min^−1^ mg^−1^ of protein in the D10 parent line to 5.0 ± 1.2 nmol min^−1^ mg^−1^ of protein in D10^γGCS^. If growth inhibition is caused by the inhibition of the parasite γGCS, elevated levels of the parasite enzyme should result in increased tolerance to BSO. Indeed, the IC_50_ value for BSO was significantly increased from 58.0 ± 1.3 µM for wild-type D10 parasites to 111.3 ± 11.1 µM for D10^γGCS^ mutant parasites (*P* < 0.001, Student's unpaired *t*-test) (Fig. 5C), which is in good agreement with the increased level of γGCS activity.

Surprisingly, no differences of GSH levels between wild type and D10^γGCS^ were found (Fig. 5D, white bars). Moreover, incubation of both parasite lines with 50 µM BSO for 2 h resulted in a similar reduction of GSH level (D10 from 832 ± 50 to 138 ± 41 nmol/10^10^ cells and D10^γGCS^ 757 ± 85 to 199 ± 74 nmol/10^10^ cells; Fig. 5D, black bars; *P* < 0.001, One-way ANOVA with Newman–Keuls post test).

### Elevated levels of γGCS lead to a decrease in GR abundance and enhanced efflux of the peptide

**Figure 6:**
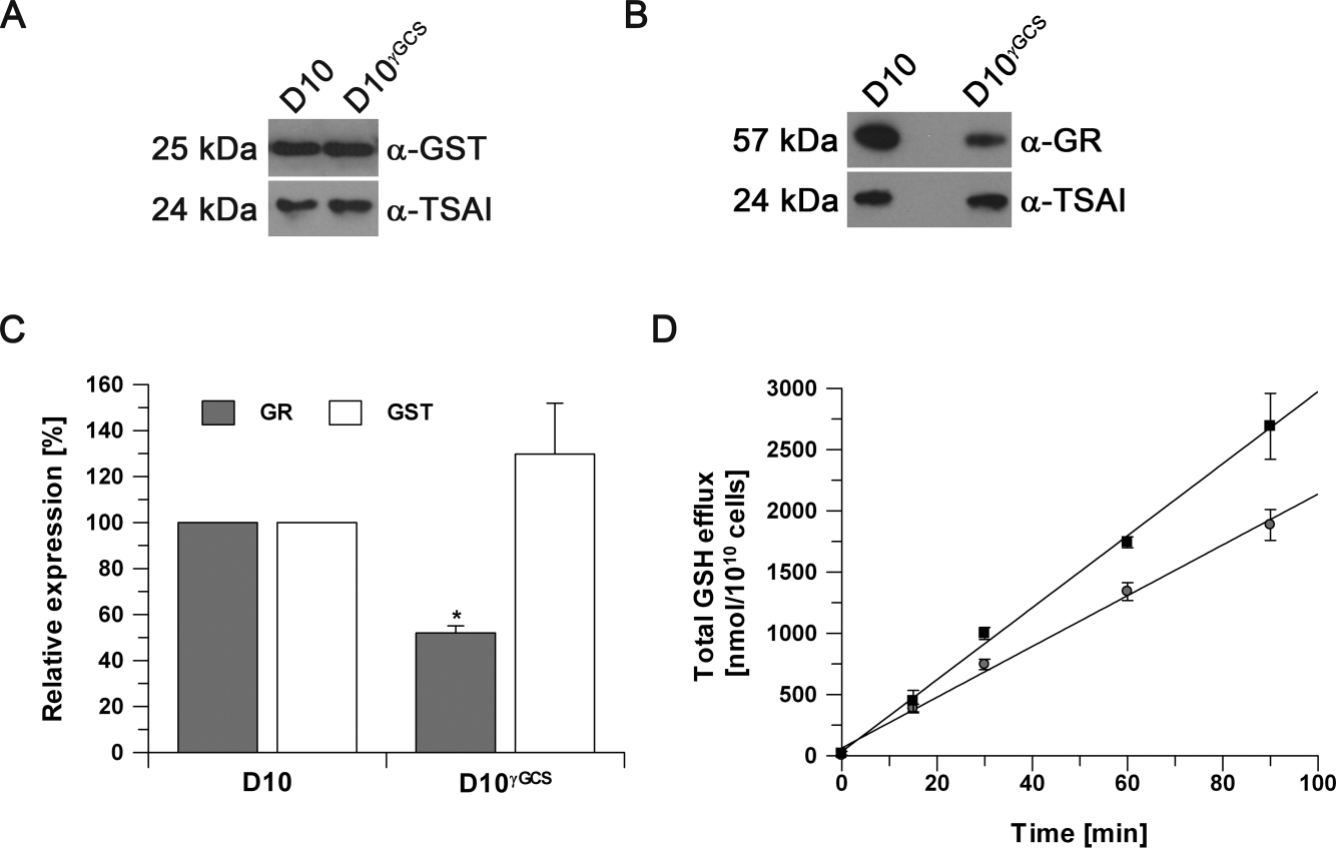
Regulation of GSH levels by GR activity and GSSG efflux. A. Western blot for GST expression using anti-*P. falciparum* GST antibody (α-GST, rabbit, 1:5000) and anti-*P. falciparum* 1-Cys peroxiredoxin antibody (α-TSAI, rabbit, 1:120 000) as loading control. B. GR expression level using anti-GR antibody (α-GR, rabbit, 1:30 000) and anti- *P. falciparum* 1-Cys peroxiredoxin (α-TSAI, rabbit, 1:120 000) as loading control. C. Relative expression of GST and GR in D10^γGCS^ normalized to expression in wild-type D10 parasites. Results are means of 3 to 4 independent determinations ± SEM (*P < 0.05, unpaired Student's *t*-test). D. Efflux of total GSH/GSSG from D10 (●) and D10^γGCS^ (▪). The efflux rate was significantly higher in D10^γGCS^ (1844 ± 123 nmol h^−1^/10^10^ cells) than in wild-type D10 (1239 ± 74 nmol h^−1^/10^10^ cells) (unpaired Student's *t*-test, *P* = 0.01).

One potential reason as to why GSH levels are not elevated in D10^γGCS^ and why BSO does not have a differential effect on parasite GSH levels during a short-term incubation, may be differences in the regulation of GSH metabolism. Other enzymes influencing GSH levels, apart from those involved in GSH biosynthesis are GR and GST. The protein levels of both enzymes were determined by Western blotting (Fig. 6A and B) and subsequent densitometric analyses (Fig. 6C). While the levels of GST were not significantly altered in D10^γGCS^ compared with D10 (Fig. 6A and C), the amount of GR was significantly reduced by 50% in D10^γGCS^ (Fig. 6B and C; Student's unpaired *t*-test; *P* < 0.001). A decrease in GR protein is likely to diminish the ability of the parasites to reduce GSSG to GSH and may result in an enhanced efflux of GSSG to maintain the intracellular redox balance. To test this hypothesis we determined the amount of total GSH that the parasites excreted into the culture medium over a 90 min period (Fig. 6D). Indeed the efflux rate increased from 1239 ± 74 nmol h^−1^/10^10^ cells to 1844 ± 123 nmol h^−1^/10^10^ in D10^γGCS^ (Student's unpaired *t*-test, *P* = 0.01) compared with that determined in D10. Together these data explain why D10^γGCS^ do not contain higher levels of GSH despite displaying elevated γGCS activity because they concomitantly loose more of the peptide attributable to an increased efflux in comparison with wild-type parasites.

## Discussion

Glutathione, a vital low molecular weight thiol in most organisms, is synthesized in two consecutive steps catalysed by γGCS and GS in *P. falciparum* (Lüersen *et al*., 1999; Meierjohann *et al*., 2002a), similar to other organisms. In this study we have attempted to generate mutant *P. falciparum* lacking the ability to synthesize GSH *de novo*. GSH biosynthesis is not essential in a variety of organisms such as *Escherichia coli* (Bouter *et al*., 1988; Lee *et al*., 2001), *Saccharomyces cerevesiae* (Lee *et al*., 2001), *Candida albicans* (Baek *et al*., 2004) and *T. brucei* (Huynh *et al*., 2003), as long as the tripeptide is provided in sufficiently high concentration in the extracellular environment. Interestingly, *S. cerevesiae* mutants lacking *gs* are viable even when they are not supplemented with GSH and can survive by using the GSH precursor γ-glutamylcysteine (γGC) as a substitute (Grant *et al*., 1997). Generally, organisms lacking a functional GSH biosynthetic pathway exhibit increased susceptibility towards abiotic stress and eukaryotic cells are particularly prone to damage of their mitochondria (Martensson and Meister, 1989; Grant *et al*., 1996; Lee *et al*., 2001; Lash, 2006).

Recently, it was reported that the intra-erythrocytic stages of the rodent malaria species *P. berghei* are also able to grow and differentiate into gametocytes without a functional GSH biosynthesis pathway (Vega-Rodriguez *et al*., 2009) and it is likely that these *γgcs* null mutants obtain GSH from their host as low levels of the peptide are still detectable in the knockout lines. However, sexual development of *P. berghei* is stopped in the insect where mitochondrial integrity is compromised due to the loss of γGCS function (Vega-Rodriguez *et al*., 2009).

In contrast to these findings, this study suggests that GSH biosynthesis is crucial not only for the development of the parasites in the insect vector but that it also has a vital function for intra-erythrocytic growth of the human malaria parasite *P. falciparum*. A loss of γGCS or GS function was not compensated by elevated levels of GSH in the culture medium; even 1 mM GSH, which is well above the concentration found in mammalian plasma and serum (Wendel and Cikryt, 1980; Jeevanandam *et al*., 2000; Scibior *et al*., 2008) does not facilitate the disruption or deletion of the genes involved in the biosynthesis of GSH. This is not due to refractory gene loci as the *γgcs* and *gs* loci were both targeted with control knock-in constructs, supporting our hypothesis that GSH biosynthesis has a vital function for *P. falciparum*. One reason for this absolute requirement in *P. falciparum* may be their inability to obtain sufficiently high amounts of GSH from their environment to compensate for the loss of GSH biosynthesis function. This is corroborated by a recent study by Raj and colleagues that showed a rate of GSH uptake of 12 pmol h^−1^/10^10^ (Raj *et al*., 2009), which, although higher than the uptake determined in this study, still is not sufficiently high to compensate for the substantial loss of GSH from the parasites during *in vitro* growth. It was determined that the parasitized RBC excrete 1239 ± 74 nmol GSH h^−1^/10^10^ cells. This has also been seen in previous studies corroborating our observation (Atamna and Ginsburg, 1997; Ayi *et al*., 1998; Lüersen *et al*., 2000). These results emphasize the necessity for *P. falciparum* to constantly generate GSH *de novo* in order to maintain adequate levels of the tripeptide. In fact the rate of GSH biosynthesis is higher than the rate of GSSG efflux from infected RBC (γGCS activity was determined to be 80–140 nmol min^−1^/10^10^ cells) (Lüersen *et al*., 2000; Meierjohann *et al*., 2002b) guaranteeing an adequate GSH homeostasis in *P. falciparum in vitro*. A potential reason as to why the parasitized RBC excrete GSH at such a high rate may be the requirement and relative cost of the GR cofactor NADPH needed to maintain GSH in its reduced form. However, this remains subject to further investigations, which are beyond the scope of this study.

Furthermore, *P. berghei* preferentially infects reticulocytes, (Janse *et al*., 1989) which suggests that different host cell preference may influence the ability of these parasites to survive without a functional GSH biosynthesis. Unlike mature erythrocytes, reticulocytes likely still have a functional γ-glutamyltranspeptidase, which would supply the infected cell with the ability to take up GSH from host plasma. This ability may give *P. berghei* a safety net that provides them with adequate amounts of GSH from their host during intra-erythrocytic development, which is lost in the mature erythrocytes that *P. falciparum* preferably infects (Meister *et al*., 1981). However, the GSH concentration in mature erythrocytes is in the mM range (Griffith, 1981), and it is surprising that *P. falciparum* appear not to use this rich source of the tripeptide. Even if GSH is taken up from the host cell into the digestive vacuole through phagocytosis it is likely digested into its amino acid components. These then individually enter the parasite cytosol, but in the absence of a functional GSH biosynthesis the parasites cannot utilize the amino acids to rebuild the tripeptide.

This study also provides evidence that in contrast to *S. cerevisiae*, *P. falciparum* is unable to exploit γGC as a substitute for GSH because it was also impossible to delete *P. falciparum gs*. Even though γGC itself has reducing properties, GSH-dependent enzymes such as GSTs, glutaredoxins and glyoxalases in *P. falciparum* are likely to be unable to make use of the dipeptide as a reducing cofactor. Another problem may be that γGC-disulphide is not efficiently reduced by GR, which would result in an imbalance of the intracellular redox homeostasis.

The role of γGCS in *P. falciparum* was further investigated using the irreversible inhibitor of the enzyme BSO (Griffith, 1982). Overexpression of γGCS in the parasites resulted in an increased IC_50_ value for BSO, implying that the inhibition of the parasite enzyme is the cause for the observed anti-plasmodial effect. Presumably the suicide inhibitor is sequestered by elevated levels of the target protein in D10^γGCS^, indicated by the increased γGCS activity determined in mutant parasites. However, the GSH levels remained unchanged in D10^γGCS^, which is attributable to the reduction of GR and the increased efflux of the tripeptide.

When the parasites were incubated for 2 h with BSO (rather than 72 h as in the IC_50_ experiment), mutant and wild-type parasites displayed a similar loss of GSH. One might expect a less pronounced loss of GSH from D10^γGCS^ in comparison with wild-type, because of elevated levels of γGCS. However, as efflux is increased, BSO inhibition of γGCS is likely to reduce GSH biosynthesis rate below the efflux rate, resulting in a similar decrease of GSH in both parasite lines. The increased IC_50_ value for BSO of D10^γGCS^ seems to contradict this interpretation. However, over a longer term of incubation with BSO it is well possible that D10^γGCS^ parasites reduce their loss of GSH possibly by upregulating GR activity or downregulating efflux of the tripeptide. Alternatively, containing multiple copies of the *γ-gcs* gene under the control of a strong promoter may allow γGCS activity to recover faster because of higher levels of re-expression of the protein.

Overall our data demonstrate that the parasites regulate their intracellular GSH levels tightly to guarantee an adequate GSH/GSSG redox homeostasis. Major regulatory processes include GSSG reduction, GSSG efflux and GSH biosynthesis, which is in agreement with reverse genetic studies in *P. berghei* that have also indicated a role for GR in regulating GSH levels (Buchholz *et al*., 2010; Pastrana-Mena *et al*., 2010).

There is mounting evidence for substantial differences between the requirements of human and rodent *Plasmodium* species regarding their antioxidant defences. Reports in *P. berghei* show that in the rodent parasite *γgcs*, *gr* and *thioredoxin reductase* are dispensable during erythrocytic development (Vega-Rodriguez *et al*., 2009; Buchholz *et al*., 2010; Pastrana-Mena *et al*., 2010), while studies in *P. falciparum* suggest that thioredoxin reductase (Krnajski *et al*., 2002) and glutathione biosynthesis (this work) are essential for parasite survival during RBC development. Our data support the hypothesis that the level of redundancy between the antioxidant and redox systems operating in the two *Plasmodium* species may differ fundamentally, a suggestion that requires further evaluation in the future.

## Experimental procedures

### Materials

Ancotil (5-fluorocytosine, 5-FC) was obtained from The Royal Infirmary Pharmacy, Glasgow, UK. WR99210 was a kind gift from Jacobs Pharmaceuticals (USA). Plasmids pHH1, pCC4, PfHsp865'-pDONR4/1, 3xHAc-pENTR2/3 and pCHD-3/4 were kind gifts of Professor A. F. Cowman (The Walter and Eliza Hall Institute, Melbourne, Australia) and Professor G. I. McFadden (University of Melbourne, Melbourne, Australia) (Crabb, 2002; Crabb *et al*., 2004; van Dooren *et al*., 2005; Braks *et al*., 2006; Maier *et al*., 2006). Blasticidin was purchased from Invitrogen. Antibodies used in Western blot experiments were obtained from Promega and Eurogentec. Anti-GST antibodies were a kind gift from Professor E. Liebau (University of Münster, Germany). All chemicals were purchased from Sigma UK unless otherwise stated.

### Plasmodium culture

*Plasmodium falciparum* 3D7 (The Netherlands) and D10 (Papua New Guinea) were used in this study. The parasites were cultured according to (Trager and Jensen, 1976) in RPMI 1640 supplemented with 0.5 % (w/v) Albumax II, 200 µM hypoxanthine, 20 µg ml^−1^ gentamycin (complete RPMI 1640 medium) in human erythrocytes at a haematocrit between 2.5% and 5%. Parasite cultures were maintained under low oxygen pressure (1% oxygen, 5% CO_2_ and 94% N_2_) at 37°C. Assays to determine IC_50_ values for BSO were performed according to Desjardins *et al*. (1979) using complete RPMI 1640 medium without hypoxanthine and supplementing the cultures with 5 µCi ml^−1^[^3^H]-hypoxanthine (20 Ci mmol^−1^, American Radiolabelled Chemicals, USA). Protein for Western blotting was isolated from parasites obtained after saponin lysis (Umlas and Fallon, 1971). Parasite pellets were resuspended in lysis buffer (100 mM HEPES, pH 7.4 containing 5 mM MgCl_2_, 10 mM EDTA, 0.5% Triton X-100, 5 µg RNase A, 1 mM phenylmethanesulfonyl fluoride (PMSF), 1 mM benzamidine, 2 µg ml^−1^ leupeptin. 10 µM E-64, 2 mM 1,10 phenanthroline, 4 µM pepstatin) and lysed by three cycles of freeze/thawing followed by 5 min centrifugation at 17 000 *g*. Concentration of soluble protein was determined using the Bradford assay with bovine serum albumin as a standard (Bradford, 1976). Genomic DNA was isolated from saponin isolated parasites (Umlas and Fallon, 1971) using the QiaAMP DNA mini kit according to the manufacturers recommendations (Qiagen, UK).

### Generation of transfection constructs

For the disruption of *γgcs* and *gs* genes the plasmid pHH1 (Crabb *et al*., 2004) was used. This plasmid contains a human dehydrofolate reductase (hDHFR) cassette for positive selection of transfected parasites. Fragments homologous to either *γgcs* or *gs* were cloned into the plasmid using BglII and XhoI restriction sites. These fragments were truncated at the 5′ end and lacked the ATG start codon as well as being truncated at the 3′ end and containing an artificial stop codon. Single cross-over recombination between the plasmids and the target genes result in two truncated non-functional copies, one lacking the 5′ end and start codon and one lacking the 3′ end and containing a premature stop codon. The 800 bp insert for *γgcs* (bp 181–981 of *γgcs* ORF) was amplified using the primers GCS-1 and GCS-2 (Table 1) and the 1004 bp insert for *gs* (bp 121–1125 of *gs* ORF) was amplified using the primer pair GS-1/GS-2.

**Table 1:** Primers used in this study.

Primer	Sequence
GCS-1	GCGC *AGATCT* CGTTATGATGAAAATATAATGTTTGG
GCS-2	GCGC *CTCGAG* ATCTTGTGTATTTGAACTATCATTAAC
GCS-3	GCGC *AGATCT* GATGTAATACTTGACAAAAATG
GCS-4	GCGC *CTCGAG* TTATGCACTCAGTTCGTAC
GCS-5	GCGC *CCGCGG* CGGAACGCCATTAAGCTGGGATG
GCS-6	GCGC *CTTAAG* CATGGCCAGTAATTCTTCTAGCC
GCS-7	GCGC *CCATGG* GAGCATCCAAAAGAGAAGCTTTAAC
GCS-8	GCGC *GGCGCC* GCAAGTATTGCGTATAATCCTCTCC
GCS-9	CACC ATGGGTTTTCTAAAAATCGGAACG
GCS-10	TGCACTCAGTTCGTACATTTTTTTTGC
GS-1	GCGC *AGATCT* GATATGATTGCTTTTTTGAATAC
GS-2	GCGC *CTCGAG* GTCGTTCAAATCGATAAGAAG
GS-3	GCGC *AGATCT* CATGAAGATGATTTTAATAGTTTTG
GS-4	GCGC *CTCGAG* CTCAATGTTCAGTTAAAAAAAAAG
GS-5	GCGC *CCGCGG* GTCCCAATGGAAAGAATGAATATC
GS-6	GCGC *CTTAAG* CGATTAGTCATATAATCAGATCTTCC
GS-7	GCGC *CCATGG* GCCTTACAAGTAGACCCATCTC
GS-8	GCGC *GGCGCC* CCAAGCTTGATATACCACAAATGG

Restriction sites within the primer sequences used for directional cloning are indicated in italics.

For knockout of *γgcs* and *gs* by double cross-over recombination the plasmid pCC4 was used (Braks *et al*., 2006; Maier *et al*., 2006). The constructs in pCC4 contained one region homologous to the 5′ end of the respective target gene in the multiple cloning site upstream of the selectable marker cassette and a second insert homologous to the 3′ region of the respective gene downstream of the selectable marker cassette. The 485 bp 5′ fragment of *γgcs* (bp 18–502 of *γgcs* ORF) was amplified with primer pair GCS-3/GCS-4 (Table 1), which contained SacII and AflII restriction sites respectively. The 522 bp 3′ fragment of the gene (bp 2549–3070 of *γgcs* ORF) was amplified using GCS-5/GCS-6 (Table 1) containing an NcoI and NarI restriction site respectively. Similarly, pCC4-*gs* was generated using primer pair GS-3/GS-4 to amplify a 480 bp 5′ region of the gene (bp 65–542 of *gs* ORF) and GS-5/GS-6 to amplify a 480 bp 3′ region of the gene (bp 1468–1942 of *gs* ORF). The fragments were restricted and subcloned into pCC4 previously cut with the respective enzymes.

Knock-in control constructs were generated by cloning a homologous region corresponding to the 3′ ends of *γgcs* and *gs* into the plasmid pHH1 (Crabb *et al*., 2004). The 1323 and 1007 bp fragments of *γgcs* and *gs* were amplified using the primers GCS-7/GCS-8 and GS-7/GS-8, respectively (Table 1). The primers introduced a BglII and an XhoI site at the 5′ and 3′ ends of the PCR products, respectively, and allowed directional cloning into the pHH1 plasmid. Single cross-over recombination of the knock-in plasmid with the respective gene locus results in a functional copy of *γgcs* and *gs* and a non-functional pseudogene downstream of the functional copy. Recombination with the target gene will however introduce an artificial 3′ UTR (*P. berghei* dihydrofolate reductase/thymidylate synthase 3′ UTR) downstream of the functional gene copy.

To express *γgcs* from an extrachromosomal copy to elevate intracellular γGCS levels, the full-length gene was amplified using GCS-9/GCS-10 (Table 1) and the PCR product was subcloned into the pENTR/D-TOPO vector (Invitrogen). To introduce a promoter to the expression cassette, the PfHsp86-5′-pENTR/1 entry clone was used (van Dooren *et al*., 2005) and the gene was C-terminally tagged with an (HA)_3_-tag using 3xHAc-pENTR2/3 in the LR clonase reaction (Invitrogen). The destination plasmid used was pCHD-3/4 (van Dooren *et al*., 2005).

All plasmid sequences were verified by nucleotide sequencing (The Sequencing service, University of Dundee).

### Transfection and selection of parasites

Before transfection parasite cultures were synchronized according to Lambros and Vanderberg (1979). Ring stage infected erythrocytes at a parasitaemia of 4–7% were washed in cytomix (120 mM KCl, 0.15 mM CaCl_2_, 2 mM EGTA, 5 mM MgCl_2_, 10 mM K_2_HPO_4_/KH_2_PO_4_ pH 7.6, 25 mM HEPES pH 7.6) (Crabb and Cowman, 1996; Crabb *et al*., 2004) and 200 µl of erythrocytes was mixed with 70–100 µg of plasmid DNA resuspended in 400 µl cytomix. The cells were electroporated at 310 V and 950 µF using a Gene Pulser Xcell electroporator (Bio-Rad). Transfected parasites were then placed into 10 ml pre-warmed complete RPMI 1640 medium and the haematocrit adjusted to 5%. Six to 8 h after transfection the culture medium was changed and the appropriate selection drug was added to the cultures. Transfectants were visible in Giemsa stained thin smears 4–6 weeks after transfection. Transfected parasites were cloned by limiting dilution according to Kirkman *et al*. following integration of the plasmids (Kirkman *et al*., 1996).

### Southern blotting

To analyse the genotypes of the transfected parasite lines diagnostic Southern blotting was performed. One µg of genomic DNA was subjected to digestion using diagnostic restriction enzymes over night at 37°C and the digested DNA was subsequently separated on a 0.8% agarose gel before blotting onto nitrocellulose using a VacuGene XL blotting apparatus (GE HealthCare). Probes corresponded to the inserts of the respective plasmids used for transfection and were labelled with thermostable alkaline phosphatase using the Gene images AlkPhos Direct Labeling and Detection kit (GE HealthCare, UK) following manufacturer's instructions. Blots were probed over night at 55°C according to manufacturer's instructions before washing and the hybridization patterns were determined by exposure to autoradiography films (Kodak) from 1 to 16 h.

### Western blotting

Ten to 20 µg of parasite protein extracts were separated by SDS-PAGE under reducing conditions before proteins were transferred to nitrocellulose using a Transblot Semidry transfer system (Bio-Rad) according to manufacturer's instructions. The primary mouse anti-HA, rabbit anti-GR antibodies and rabbit anti-1-Cys peroxiredoxin antibodies (all Eurogentec) were used at 1:1000, 1:30 000 and 1:120 000 dilutions, respectively. The rabbit anti-GST antibodies were used at 1:5000 dilution. The secondary anti-mouse-HRP conjugate or anti-rabbit-HRP conjugate (Promega) antibodies were used at 1:10 000 dilutions. The protein bands reacting with the antibodies were detected using the Millipore Immobilon Western Blot detection kit and visualized by exposing blots to autoradiography films (Kodak).

### Purification of trophozoite-infected RBC using magnet activated cell sorting (MACS) columns

Trophozoite-infected RBC were resuspended in 10 ml Earle's balanced salt solution (EBSS; Thermo HyClone) and applied to a CS MACS column placed into the magnetic field of a VarioMACS separator (Miltenyi Biotech). Trophozoite-infected RBC were retained while uninfected RBC were washed through the column with 50 ml EBSS. The infected RBC were eluted by removing the column from the magnetic field and washing it with 30 ml EBSS using a syringe. The purified trophozoite-infected RBC were centrifuged for 5 min at 700 *g*, 4°C and the supernatant was removed. The purified cells had a parasitaemia of 85–90% and were carefully resuspended in EBSS at a cell number appropriate for the following applications.

### GSH uptake assays

MACS-purified infected RBC were resuspended in pre-warmed EBSS at 2 × 10^8^ cells ml^−1^. As a control non-infected RBC were used at the same cell count. An equal volume of EBSS containing only 48 nM [^3^H]-GSH (2 µCi ml^−1^, American Radiolabelled Chemicals, USA) or 48 nM [^3^H]-GSH and 6 mM cold GSH was added and carefully mixed, giving final concentrations of 24 nM [^3^H]-GSH and 3 mM cold GSH. The cells were incubated in a water bath at 37°C and at various time points 200 µl samples corresponding to 2 × 10^7^ RBC were removed and cells were immediately separated from the uptake solution by centrifugation through 300 µl dibutylphthalate as described in Biagini *et al*. (2005) and Quashie *et al*. (2008). The uptake solution was removed and the tube walls carefully rinsed with H_2_O and wiped with a clean tissue. The oil was removed and cells were lysed in 100 µl H_2_O. To bleach haemoglobin 50 µl H_2_O_2_ were added followed by the addition of 50 µl acetic acid. Three millilitres of scintillation cocktail were added and the samples were counted the following day in a Wallac Trilux MicroBeta scintillation counter.

### GSH efflux

MACS-purified trophozoite-infected RBC were resuspended in 350 µl EBSS and the cell number was determined using a haemocytometer. GSSG-efflux was analysed by incubating purified infected RBC at 37°C in EBSS removing 50 µl of the cell suspension at various time points. The cells were pelleted by centrifugation at 400 *g* for 2 min in a benchtop centrifuge and 25 µl of the supernatant was used for derivatization of thiols as described below.

### Determination of γGCS activity

γGCS activity was determined as previously described (Lüersen *et al*., 2000) with minor modifications. The 100 µl reaction contained 0.1 M Tris-HCl buffer, pH 8.0, 20 mM MgCl_2,_ 2 mM EDTA, 10 mM L-α-aminobutyrate, 10 mM ATP, 5 mM creatine phosphate, 1 unit of creatine phosphokinase, 10 mM [U-^14^C] glutamate (1 µCi, 270 mCi mmol^−1^) and 30 µg of parasite lysate obtained from wild type and mutant parasites after saponin lysis. The reaction was incubated for 45 min at 37°C before the reaction was stopped by addition of 10 µl of 2 M HClO_4_ and heating to 95°C for 3 min. After centrifugation at 13 000 *g* for 5 min, 10 µl of the supernatant was applied to thin layer chromatography plates (cellulose F plastic sheets, 20 × 20 cm; 0.1 mm thickness; Merck). The reaction products were separated using butan-1-ol/acetic acid/water (12:3:5, by volume) for 24 h. The plates were dried and exposed to film for at least 24 h to visualize the reaction product γ-glutamylaminobutyrate as a spot on the film. The radioactive spots were cut out, transferred into 3 ml scintillation fluid and the radioactivity determined in a Wallac Trilux MicroBeta liquid scintillation counter. The specific activity was calculated according to amount of parasite protein and is given in γ-glutamylaminobutyrate generated in nmol min^−1^ mg^−1^ of parasite protein.

### Determination of GSH level

Parasites were isolated from 20 ml of synchronized culture (∼ 2–5 × 10^7^ parasites) by saponin lysis and GSH levels were determined by HPLC as described by Williams *et al*. (Williams *et al*., 2009). The cells were washed once with EBSS before lysis of RBC with 0.15% saponin (for 5 min on ice), followed by three washes with EBSS at 4°C. The parasite number was determined using a haemocytometer and the parasites were incubated for 45 min at room temperature in 50 µl of derivatization buffer (40 mM *N*-[2-hydroxyethyl]-piperazine*-N*′[3-propanesulfonic acid], 4 mM diethylenetriamine pentaacetic acid, pH 8.0), containing 0.7 mM tris(2-carboxyethyl)phosphine to allow reduction of thiols. 50 µl of 2 mM monobromobimane in ethanol were added and samples heated to 70°C for 3 min. After a brief cooling period extracts were deproteinized for 30 min on ice by addition of 100 µl 4 M methanesulfonic acid, pH 1.6. Precipitated protein was removed by centrifugation in an Eppendorf benchtop centrifuge for 40 min at 16 000 *g* at 4°C. The supernatant was analysed by HPLC as described by Williams *et al*. (Williams *et al*., 2009).

### Statistical analyses

For statistical analyses GraphPadPrism software was used. Results are represented as means ± standard deviation (SD) or standard error of the mean (SEM). Data were analysed by unpaired Student's *t*-test for two groups or by one-way ANOVA and Newman–Keuls post test for multiple groups.
