# Supplementary material for: Dissecting the role of glutathione biosynthesis in Plasmodium falciparum
Source: Mol Microbiol. 2012 Jan;83(2):304–18. doi: 10.1111/j.1365-2958.2011.07933.x (PMC3321222; doi:10.1111/j.1365-2958.2011.07933.x)
Supplement: Supplementary file 1 [file mmi0083-0304-SD1.pdf]

Supplementary Material- Patzewitz et al., 2011

Supplementary Figure 1: Co-transfection of pCC4-*γgcs* and pCHD-*γgcs(HA)*$_{3}$.

(A) Schematic representation of the endogenous *γgcs* locus, the pCC4-*γgcs* and pCHD-*γgcs*(HA)$_3$ plasmids and the recombined locus following double crossover recombination between the endogenous gene and the pCC4-*γgcs* plasmid. NdeI restriction sites and the expected fragment sizes are indicated. (B) Southern blot analysis using a *γgcs* specific probe on non-transfected wild type parasites (lane 1) and parasites co-transfected with both plasmids (lane 2). The endogenous 3.7 kb *γgcs* band is detected in both parasite lines and the 7.3 kb and 0.7 kb bands corresponding to pCC4-*γgcs* are visible in the transfected parasites. In addition a band not corresponding to any of the expected bands for either the pCHD-*γgcs(HA)*$_3$ expression plasmid or an integration event is visible in the transfected parasites below the endogenous *γgcs* band. (C) Southern blot analysis using probes specific for the selectable markers Bla and hDHFR. No bands are visible in the non-transfected wild type (lane 1). In the transfected parasite line (lane 2) a band corresponding to the 7.3 kb fragment of pCC4-*γgcs* containing the selectable marker is detected as well as the same unexpected band that was detected with the *γgcs* specific probe. (D) Diagnostic PvuII digest of plasmids isolated from genomic DNA of the transfected line. The expected sizes for pCC4-*γgcs* are 3.6 kb and 4.8 kb and for pCHD-*γgcs(HA)*$_3$ 8.3 and 2.4 kb.

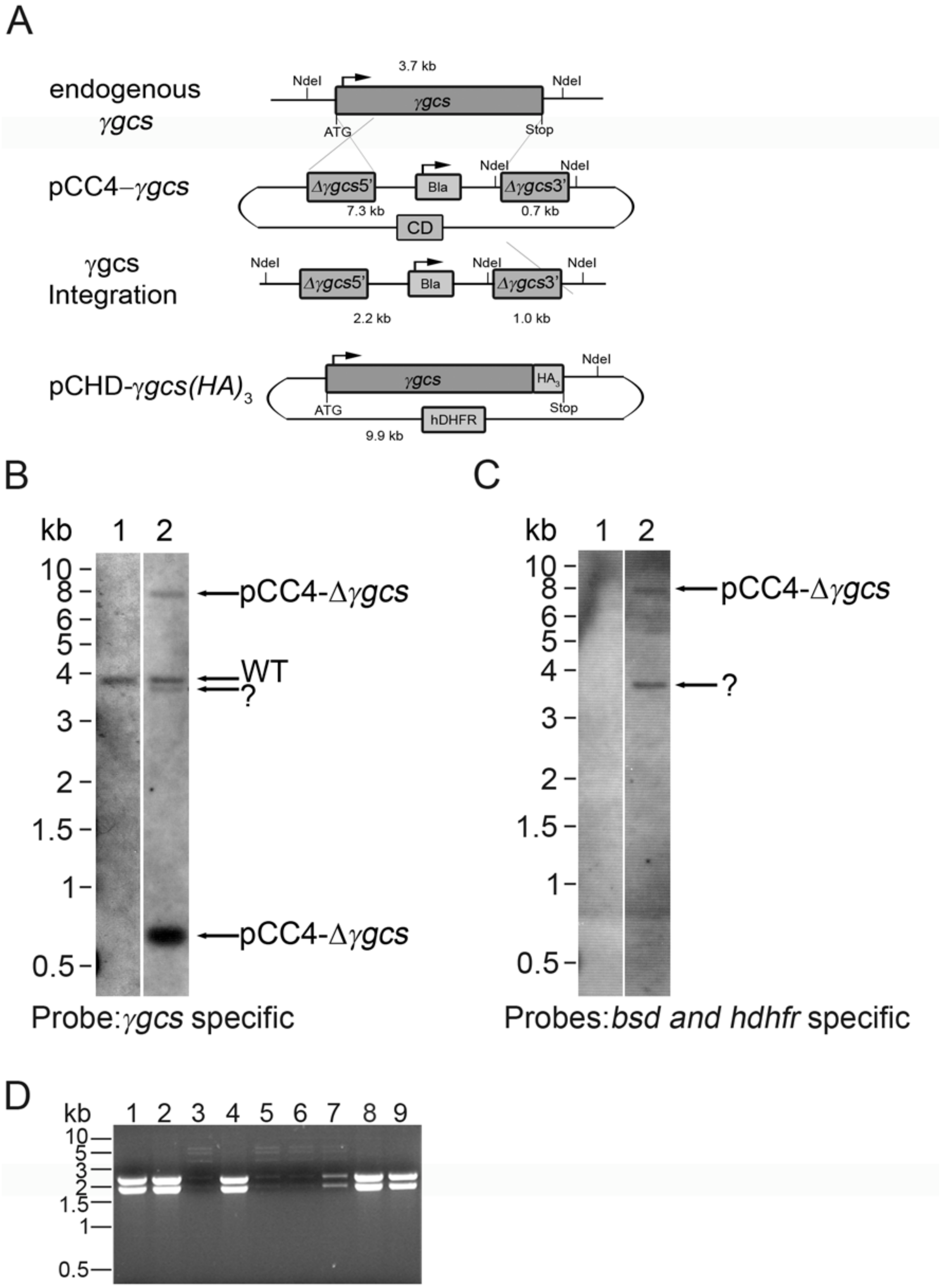
A
endogenous
γgcs
3.7 kb
NdeI
γgcs
NdeI
ATG
Stop
pCC4–γgcs
Δγgcs5'
Bla
NdeI
Δγgcs3'
NdeI
7.3 kb
0.7 kb
CD
γgcs
Integration
NdeI
Δγgcs5'
Bla
NdeI
Δγgcs3'
NdeI
2.2 kb
1.0 kb
pCHD-γgcs(HA)3
γgcs
HA3
NdeI
ATG
Stop
hDHFR
9.9 kb
B
kb
1
2
10
8
6
5
4
3
2
1.5
1
0.5
pCC4-Δγgcs
WT
?
pCC4-Δγgcs
Probe:γgcs specific
C
kb
1
2
10
8
6
5
4
3
2
1.5
1
0.5
pCC4-Δγgcs
?
Probes:bsd and hdhfr specific
D
kb
1 2 3 4 5 6 7 8 9
10
5
3
2
1.5
1
0.5

Supplementary Figure 2: Long term uptake of GSH

iRBC were cultured in the presence of 24nM [^{3}H]-GSH for up to 4 h. At the indicated time points (0 h, 2 h and 4 h), parasites were isolated from RBCs by saponin lysis at 4 °C to stop GSH transport and the parasites were processed for scintillation counting as described for iRBC (see “GSH uptake assays”). Over a 4 h period, the amount of [^{3}H]-GSH increased with an apparent uptake rate of 1-2 pmol / h / 10^{10} cells.

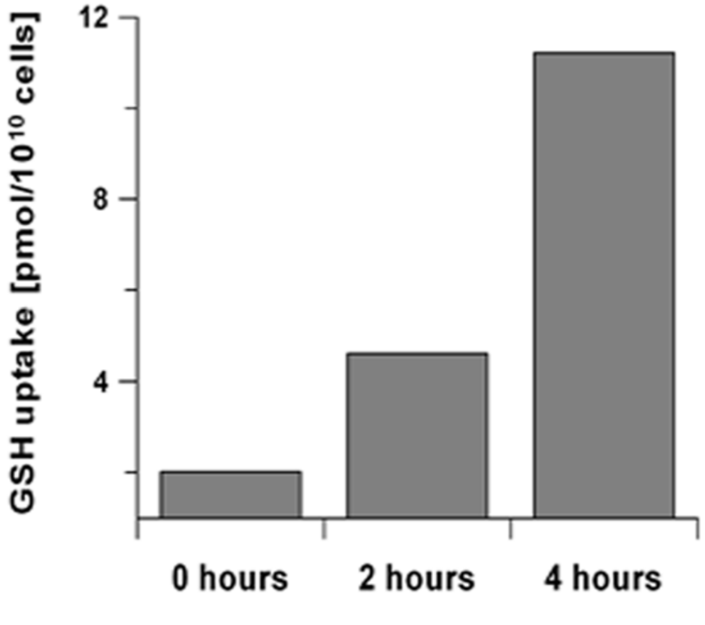
GSH uptake [pmol/10^10 cells]
12
8
4
0 hours
2 hours
4 hours
